# An Overview of New PAT Freeze-Drying Methods Based on Shelf Temperature Inlet/Outlet Difference or Chamber/Condenser Pressure Difference: Theory and Practical Use

**DOI:** 10.3390/pharmaceutics17101277

**Published:** 2025-09-30

**Authors:** Jean René Authelin

**Affiliations:** Centre de Recherches de Vitry, Sanofi, 94400 Vitry sur Seine, France; jean-rene.authelin@sanofi.com

**Keywords:** freeze drying, process analysis technology, software sensor

## Abstract

**Background/Objectives:** Recently, new methods of monitoring sublimation flow during freeze-drying operations have been proposed. They are based either on measuring the difference between the temperature of the heat transfer liquid at the inlet and outlet of the shelves (Δ*T*) or the difference between the chamber pressure and the condenser pressure (Δ*P*). In this article, we briefly explain the two methods and review their main applications. **Methods:** Multiple pilot or commercial-scale freeze dryers were used. The inlet and outlet shelf temperature or the capacitance pressures of the chamber and condenser were measured. **Results:** Δ*T* and Δ*P* methods can be implemented in most recent freeze dryers to monitor the sublimation flow. Both methods provide very consistent results and are also comparable to Tunable Diode Laser Absorption System (TDLAS) measurements. The methods can be used for different purposes: calculating the heat transfer coefficient (*Kv*) distribution from the mass flow curve and estimating the average product temperature and the product temperature range. Furthermore, these methods can be used as a measure of success for transferring the process from the lab to the industrial scale, or from one plant to another, or demonstrating the shelf-to-shelf homogeneity. Finally, the Δ*T* method is able to detect the ice nucleation during the freezing step. **Conclusions:** The Δ*T* and Δ*P* methods are bringing a new, easy-to-implement, cost-effective, and versatile tool to the freeze-drying study toolbox.

## 1. Introduction

Process Analysis Technologies (PATs) are an indispensable element of the Quality by Design (QbD) methodology applied to pharmaceutical process development [1]. “ICH Q8 (R2) identifies the use of PAT to ensure that the process remains within an established design space”. Freeze-drying-specific PATs have been developed [2,3]. Typical PATs include (wired or wireless) product temperature sensors, which allow for the determination of the product temperature history and the calculation of the heat transfer coefficient (*Kv*) or the cake resistance (Rp) [4]. The use of product temperature probes is ubiquitous at the lab scale and common for technical batches at large scale, but is most often prohibited in Good Manufacturing Practices (GMP) conditions, due to the risk of contamination. The comparison of the pressure indicated by the capacitance pressure probe and the Pirani pressure probe allows for the detection of the end of the primary drying. The use of the Pirani/capacitance pressure ratio is available in most freeze dryers, for technical and GMP use. It is clearly considered part of the best practices [5]. Manometric Temperature Measurement (MTM) [6,7] is a software sensor method to estimate the product temperature. It is based on the chamber pressure rise when the chamber is isolated from the vacuum; therefore, it is quite invasive. This method is reserved for some laboratory equipment. The heat flux method consists of inserting a very thin heat flow sensor between the shelf and the vial. This sensor converts the heat flow into an electromotive force (Seebeck effect). It allows for measuring heat flow due to ice nucleation and crystallization [8] or to sublimation [3]. This technique is also reserved for lab-scale experiments. The Tunable Diode Laser Absorption System (TDLAS) [6,9,10] is an optical system measuring simultaneously the velocity by the Doppler effect and the concentration of the vapor flowing through the chamber to the condenser pipe by laser absorption. The vapor mass flow is then calculated. Although TDLAS is a very powerful technique, it is mostly used at the laboratory scale due to the complexity and cost of retrofitting the system at the commercial scale. A new method to measure the mass flow based on the weighing of the shelves themselves has been recently proposed. It may be applicable in industrial conditions, with some equipment modification [11].

As a conclusion, in practice, in GMP conditions, the only commonly used PAT is the Pirani to capacitance pressure comparison. Very important parameters, including the product temperature or the sublimation rate, are not routinely measured in commercial production. Therefore, whereas the available PATs allow a very comprehensive characterization of the freeze-drying step at lab scale, the commercial scale appears as a black, or at least, a gray box. To mitigate this issue, we have proposed to use sensors that are already present in state-of-the-art commercial freeze dryers: the shelf inlet and outlet temperature and/or the chamber and condenser capacitance pressure [12]. Thanks to these measurements, it is possible to determine the primary drying sublimation mass flow [12] or to detect the ice nucleation [13]. Furthermore, once the sublimation mass flow is known, one may even calculate the *Kv* distribution or the distribution of product temperatures [14]. It should be noted that [12] was not the first article to consider the pressure to condenser difference. Already in 2007, Patel et al. [15] noticed the difference between the chamber and condenser pressures due to the gas flow, but did not propose to use it to calculate the mass flow. More recently, Kawazaki et al. [16] proposed using the Δ*P* signal to estimate the sublimation mass flow. The goal of this article is to review the theory behind these PATs and how they can be used in practice.

## 2. Theory

The approaches presented in this article use only the measurements from sensors generally available in recent freeze dryers: on the one hand, the measurement of the temperature of the heat transfer liquid at the inlet and outlet of the shelves, on the other hand, the capacitive pressure measured in the chamber and in the condenser. They require no investment and can be applied retrospectively if the data are recorded and can be exported to calculation software such as Excel^TM^.

### 2.1. ΔT Method

This method [12,14] is a calorimetric method that enables estimation of both the sublimation flow during the primary drying and the ice crystallization during the freezing stage. It is based on the heat balance: the heat exchanged by the heat transfer fluid (typically silicone oil) is the sum of two contributions:

1. The sensible heat linked to the temperature change of the entire apparatus (shelves, bottles, liquid, or ice in vials);
2. The heat released or absorbed by phase transitions (crystallization of ice during freezing or sublimation of ice during primary drying).

(1) $$Q_{oil}C_{p,oil}∆T_{I/O}=\Gamma \frac{d<T>}{dt}+\Delta H\frac{dM}{dt}$$

*Q_oil_* (kg/s) and *C_p,oil_* (J/kg.°C) represent the mass flow and the specific heat capacity of the silicone oil circulating through shelves, respectively. $∆T_{I/O}$ (°C) is the liquid inlet/outlet temperature difference. (J/°C) is the overall combined heat capacity of the shelves (metal) and vials (glass + water or ice). In the case of the sublimation, Δ*H* is the sublimation enthalpy (Δ*H_s_* ~2836 kJ/kg, endothermic) and *dM/dt* is the total sublimation rate (kg/h). In the case of the crystallization, ΔH is the ice crystallization enthalpy (Δ*H_c_*~−333 kJ/kg, exothermic) and *dM/dt* is the total ice crystallization rate (kg/h).

The term $\Gamma \frac{d<T>}{dt}$ accounts for the sensible heat (i.e., heating or cooling the metallic shelves and the vials). It is a dominant term when quick heating or cooling ramps (high $\frac{d<T>}{dt}$ absolute value) are applied and vanishes when the shelf temperature is constant ($\frac{d<T>}{dt}=0$). It should be noted that this term assumes that all equipment is instantaneously brought to the average temperature of the heat transfer fluid (*<T>*), which is only true to a first approximation. This can generate spurious “peaks”, which we will point out later in the article. Manipulating Equation (1), we get the sublimation (or crystallization) flux:

(2) $$\frac{dM}{dt}=\frac{Q_{oil}C_{p,oil}}{\Delta H}(∆T_{I/O}-\frac{\Gamma }{Q_{oil}C_{p,oil}}\frac{d<T>}{dt})$$

The ratio $\frac{\Gamma }{Q_{oil}C_{p,oil}}$ can be easily estimated, for example, by running a heating/cooling cycle in the absence of sublimation or crystallization (*dM*/*dt* = 0). By integration of Equation (2), we get:

(3) $$\Delta M=\int_{start\;D1}^{end\;D1}\frac{dM}{dt}dt=\frac{Q_{oil}C_{p,oil}}{\Delta H}\int_{start\;D1}^{end\;D1}(∆T_{I/O}-\frac{\Gamma }{Q_{oil}C_{p,oil}}\frac{d<T>}{dt})dt$$

where *D*1 means primary drying (should be replaced by start and end of freezing for the ice crystallization). Finally, it is not necessary to know the term $Q_{oil}C_{p,oil}$. Indeed, it is eliminated in further calculations (combining Equations (2) and (3)), and it is only necessary to know the total mass of ice (Δ*M*), which was sublimated (or crystallized). Finally, the mass flux is fully determined by:

(4) $$\frac{dM}{dt}=\frac{\Delta M}{\int_{start\;D1}^{end\;D1}(∆T_{I/O}-\frac{\Gamma }{Q_{oil}C_{p,oil}}\frac{d<T>}{dt})dt}(∆T_{I/O}-\frac{\Gamma }{Q_{oil}C_{p,oil}}\frac{d<T>}{dt})$$

### 2.2. ΔP Method

One of the simplest methods of measuring the flow rate of a gas or a liquid is to measure the pressure drop caused by the fluid flowing through a calibrated orifice. In the case of freeze-drying, the vapor flow through the pipe and the mushroom or butterfly valve creates the pressure drop (Δ*P*) between the chamber and the condenser. The method consists of correlating Δ*P* with the vapor mass flow rate, according to the equation proposed in [12]:

(5) $$\frac{dM}{dt}={\beta \left(<P>∆P\right)\;}^{\alpha }$$

α and β are constants, *<P>* is the average chamber/condenser pressure, and Δ*P* is the difference in pressure between the chamber and condenser. In [12], it was demonstrated that this relationship is exact in the laminar regime (α = 1) or in the turbulent regime (α = 0.5). In intermediate situations (for instance, if the valve works like a singular pressure drop in a turbulent regime and the pipe is experiencing a laminar regime), the relationship is an empirical correlation with 0.5 < α < 1.

One should note that the total sublimated mass (Δ*M*) is obtained by integrating Equation (5).

(6) $$∆M=\beta \int {\left(<P>∆P\right)\;}^{\alpha }dt$$

Equation (6) shows that the sublimated mass should be proportional to the integral of ${\left(<P>∆P\right)\;}^{\alpha }$. This relationship will be used to determine the α and β constants. Note that Kawazaki et al. [16] already proposed an equation equivalent to Equation (5).

## 3. Materials and Methods

All experiments related in this paper are described in detail in references [12,13,14]. Table A1 in the Appendix A gives a short summary of the different pilot and industrial freeze dryers and experimental conditions. All freeze dryers were equipped with shelf inlet/outlet temperature probes (even one temperature probe for the exit of each shelf for industrial freeze dryers) and chamber/condenser capacitance probes. Some of the examples are from real commercial production, and detailed operating conditions cannot be disclosed. All freeze dryers are equipped with shelf inlet/outlet temperature probes and chamber/condenser capacitance pressure probes, except the SMH 90 (no capacitance pressure in the condenser). All calculations were performed with Excel^TM^ or with the Sanofi internal lyo-simulation tool.

## 4. Primary Drying Sublimation

### 4.1. Data Treatment

Two sensors of temperature and pressure, even if they are well calibrated, exposed to the same conditions, provide very close values, but rarely perfectly identical ones. For example, a calibration offset of 0.1 to 1 °C for temperature sensors, or a few µbars for pressure sensors, is typical. This difference may lead to very significant errors in both the Δ*T* and Δ*P* methods if it is not corrected. In practice, we assume that the sensor offset is constant and equal to the baseline value of the signal $(∆T_{I/O}{}^{\circ}\;$or ∆P°) at the end of the sublimation step (resp. at the end of the freezing) when there is no more sublimation flow (resp. crystallization), nor change in the shelf temperature or in the chamber/condenser pressure. The baseline corrected signal is simply $∆T_{I/O}-∆T_{I/O}{}^{\circ}$ for the Δ*T* method or ∆P−∆P° for the Δ*P* method. The difference in sensor indication is not necessarily due solely to the difference in sensor calibration. For example, the oil flowing through the shelves may slightly warm up under the effect of the heat radiated from the freeze dryer walls, or the small stream of nitrogen constantly sweeping through the chamber may cause a slight drop in pressure (internal data suggest that the effect is << 1 µbar). These effects are globally taken into account in the baseline correction.

**Figure 1:**
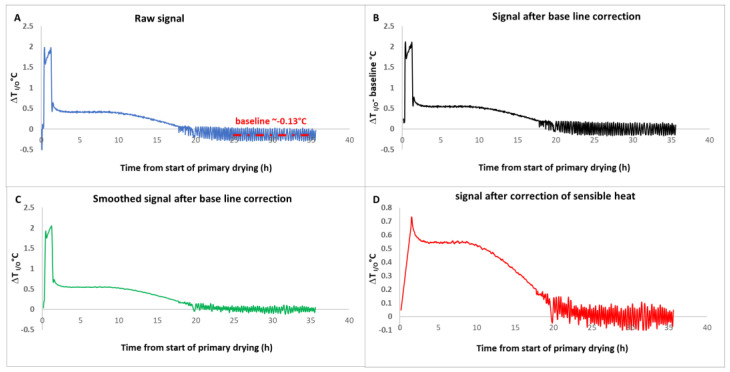
(**A**): Raw Δ*T* signal-(baseline: average signal for the period 25 and 35 h), (**B**): Δ*T* signal after baseline subtraction, (**C**): smoothed signal, (**D**): final Δ*T* signal after sensible heat subtraction based on Equation (3). Adapted from Reference [12].

After baseline correction, the Δ*T* or Δ*P* signals are often scattered, in general, because of the temperature or pressure regulation systems. Typically, we use an acquisition frequency of one point every 1 to 5 min, and we use a moving average procedure to eliminate most scattering (we use an averaging period of 10 to 20 min, depending on the case). It is only on the corrected signal that Equation (4) is applied for the Δ*T* method (or Equation (5) for the Δ*P* method). Figure 1 illustrates the complete procedure for the Δ*T* method. In Figure 1A, at a time comprised between 25 and 35 h, the signal oscillates around its baseline (~−0.13 °C). In Figure 1B, the signal has been corrected by subtracting the baseline. In Figure 1C, the baseline-corrected signal has been smoothed by applying a moving average (11 min period of averaging), which erases the oscillations without altering the shape of the signal. Finally, in Figure 1D, the impact of the sensible heat has been removed by calculating $∆T_{I/O}-\frac{\Gamma }{Q_{oil}C_{p,oil}}\frac{d<T>}{dt}$. Note that the initial small peak in the corrected Δ*T_I/O_* signal (Figure 1D) is most probably a calculation artefact due to the approximation mentioned in the theoretical part. Indeed, after the end of the initial heating ramp, when the shelf temperature reaches its plateau, it is for d<T>/dt = 0, and the equipment is still at a slightly lower temperature than the fluid, leading to some residual heat exchange and a peak in the Δ*T* signal. A more refined procedure to eliminate this peak was proposed in the supplementary information section of reference [12].

For the Δ*P* method, the initial processing of the data is identical: determination of the baseline corresponding to the offset of the pressure transducers and smoothing of the curves to eliminate fluctuations due to the pressure regulation system. Once these operations have been carried out, the quantity *<P>*Δ*P* is calculated. For the Δ*P* method, there is no equation equivalent to Equation (4), so we need to find other methods to correlate *<P>*Δ*P* to the sublimation flux.

**Figure 2:**
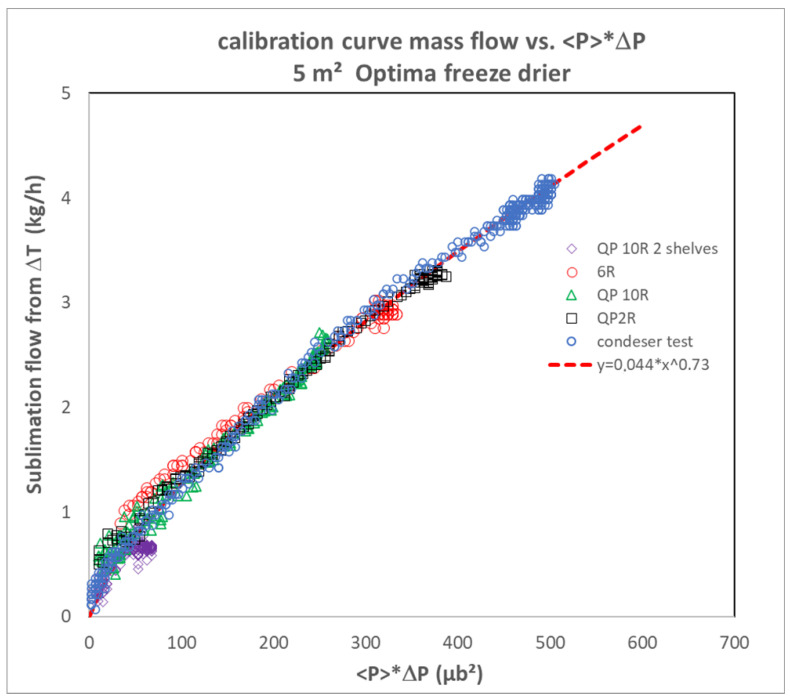
Illustration of the method of direct comparison of the mass flow to <*P*>Δ*P* adapted from [12]. 2R, 6R vials were filled with pure water (all 6 shelves). 10R vials were filled with 8% sucrose placebo. In one of the 10R vials trials, only 2 shelves were filled. Trays of water were filled (as part of condenser qualification tests). The tests with vials were operated at 133 µbar, whereas the test with water trays was operated at 100µbar. Adapted from reference [12]. See more details in [12].

Two methods are proposed by [12]. The first method consists of plotting *<P>*Δ*P* as a function of the mass flux determined based on the Δ*T* method. It is preferable to combine several sets of data acquired at various pressures and to cover a large range of flow rates (Figure 2). The second method consists of first calculating the integral $\int {\left(<P>∆P\right)\;}^{\alpha }dt$ on the basis of pressure data collected for several batches manufactured on a unique freeze dryer under different pressures and with a wide range of loads. Then, the integral is plotted against the sublimated mass of ice. The points align only for the right α value, and the slope of the line is 1/β [12].

**Figure 3:**
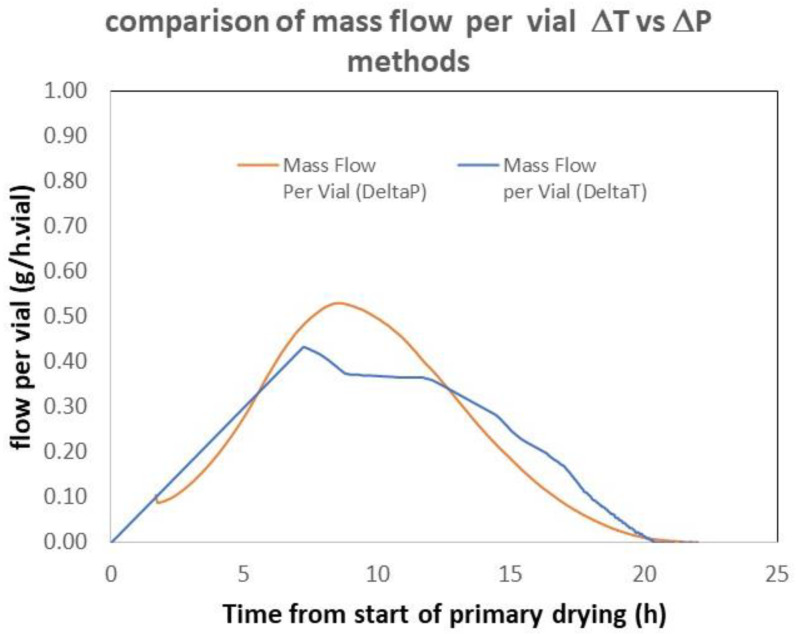
Comparison of the mass flow obtained on a 29 m^2^ commercial freeze dryer. The Δ*P* method was calibrated according to the “integral” method. Adapted from reference [12].

As they are based on completely independent measurements, the Δ*T* and Δ*P* methods are orthogonal, especially when the Δ*P* method has been calibrated by comparing the integral and the sublimed mass (second method). It is therefore interesting to compare their results. Figure 3 shows an example taken from the experimental data used in reference [12]. The results obtained using both methods are not perfectly superimposable but still consistent. One should also remember that the TDLAS and Δ*P* methods exhibited very consistent results [12].

### 4.2. Kv Distribution Determination

**Figure 4:**
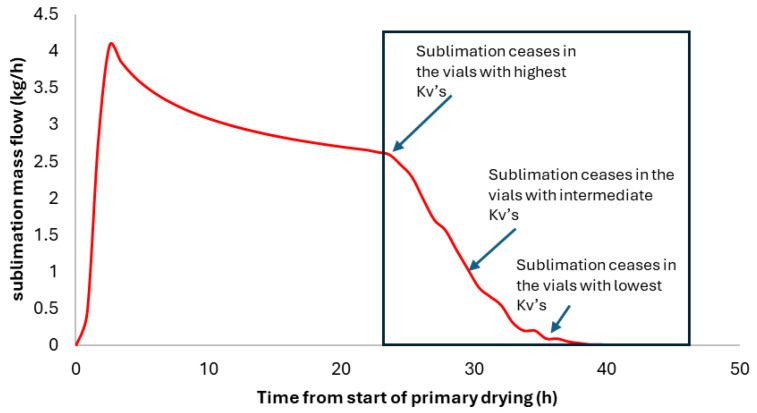
Simulated sublimation mass flow curve. The end of the curve exhibits a sigmoidal shape. The “oscillations” on the mass flow curve are due to the discretization of the *Kv* distribution in 30 classes.

In reference [14], it was shown that sublimation flux can be traced back to the distribution of *Kv* (heat transfer coefficients), regardless of the method used to measure sublimation flux. For example, the TDLAS, Δ*T*, or Δ*P* methods could be used. The basic idea is simple: if all vials had the same *Kv*, then the sublimation flow would have to stop abruptly at the point where the sublimation front descending at approximately constant speed reaches the bottom of the vial. However, the end of the flow curve usually exhibits a sigmoidal shape (Figure 4). Sublimation stops first for the vials with the highest *Kv* (typically the edge vials). As these vials are few, they cause a moderate drop in total sublimation flux, indicated by the initial dip in the sublimation flux curve. Next come most vials, corresponding to intermediate *Kv’s*, for which sublimation ceases within a relatively short time period. These vials correspond to the almost vertical part of the sigmoidal curve. The end of the flow curve corresponds to the slowest vials with the lowest *Kv* values of the distribution. Using the model developed by reference [17], it is possible to calculate the *Kv* value for a class of vials for which sublimation stops at a time “*t*”. Once the *Kv* value for that vial class has been determined, one can calculate the sublimation flux per vial in it, just before the sublimation ends. Finally, knowing the drop in total flux between times *t* and *t + dt*, corresponding to vials in the class (*Kv*; *Kv + dKv*), we can determine the number of vials in this class by dividing the total flow decline during the period by the flow per vial of this *Kv* class. By repeating this calculation over the entire sigmoidal period, we construct the *Kv* cumulative distribution curve. This distribution is fit by a bi-modal distribution model developed in [18]. Detailed equations are provided in reference [14].

**Figure 5:**
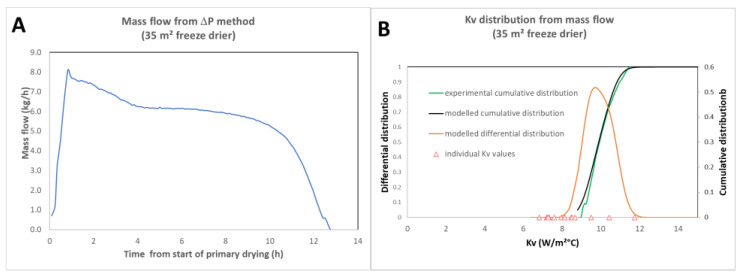
(**A**) Mass flow for a full load technical placebo lyo cycle (35 m^2^); (**B**) cumulated experimental and best fit *Kv* distributions superimposed with the modelled differential distributions. As product temperature probes were placed in some vials, individual *Kv* values (triangles) were calculated and plotted for comparison. Adapted from ref [14].

Compared with conventional methods for determining *Kv* [18,19,20] (where *Kv* is estimated by the amount of ice sublimated in hundreds of vials over a time interval during dedicated trials), this method offers several advantages. Firstly, there is no need for dedicated testing and cumbersome vial-weighing methodology. The distribution calculation can be performed directly on any batch: technical, clinical, or commercial. For *Kv* determination at commercial scale [20], this is particularly important, as the immobilization of an industrial installation for *Kv* measurements by ice sublimation is very costly. What is more, in practice, *Kv* can only be measured in a restricted sample—at most a few percent of vials—whereas the sublimation flow method offers a global view of distribution. Furthermore, the technique does not require the use of product temperature probes, although this can provide interesting additional information. Figure 5 shows the example of the sublimation mass flow measured by the Δ*P* method and the distribution calculated based on this measurement (data acquired in a large-scale freeze dryer: IMA lyofast-35 m^2^).

### 4.3. Product Temperature Estimation

Once the *Kv* distribution has been determined, the product temperature is easily calculated. We propose two methods.

On the first hand, based on the average *Kv* and the average mass flow per vial (*dm/dt*), the difference of temperature between the shelf temperature (*T_s_*) and the bottom (*T_b_*) is provided by:

(7) $$\left(T_{s}-T_{b}\right)=\frac{∆H_{s}}{K_{v}A_{v}}\frac{dm}{dt}$$

where $∆H_{s}\;$is the ice sublimation enthalpy (2836 J/g) and A_v_ is the area of the vial outer section (m^2^). As the shelf temperature is known, the bottom temperature is known as well. Similar results were obtained by using the Manometric Temperature Measurement [6] or TDLAS [9,10] or by measuring the chamber/condenser pressure difference [16].

**Figure 6:**
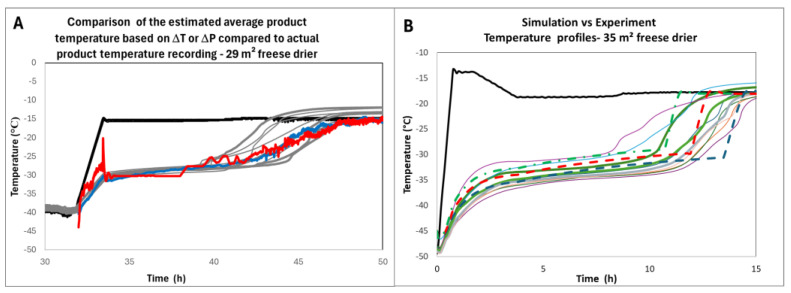
(**A**) Product temperature estimation based on Δ*T* (red) and Δ*P* (blue) methods compared to actual probe measurements (light grey) during an engineering batch of an undisclosed product; (**B**) observed and predicted temperature profile during the engineering batch of an undisclosed-different-product. Solid lines are actual temperature measurements. The green, red, and blue dashed lines are the 0.5% largest, median, and 0.5% smallest *Kv’s* [14]. Adapted from references [12] and [17]. In both graphs the black line is the shelf temperature.

On the other hand, it is possible to use the model developed by [17] based on *Kv* distributions, provided the cake resistance has been determined. The model allows for calculating the temperature in the different classes and provides the range of expected product temperature profiles. The comparison of the estimated average temperatures or temperature range with measurements taken during the manufacture of engineering batches for which temperature probes were used (Figure 6A,B) shows that the prediction is very close to the measurement. Consequently, measuring sublimation flux using the Δ*T* or Δ*P* (or TDLAS) methods enables a software sensor to be set up for product temperature estimation.

### 4.4. Lyo Homogeneity

**Figure 7:**
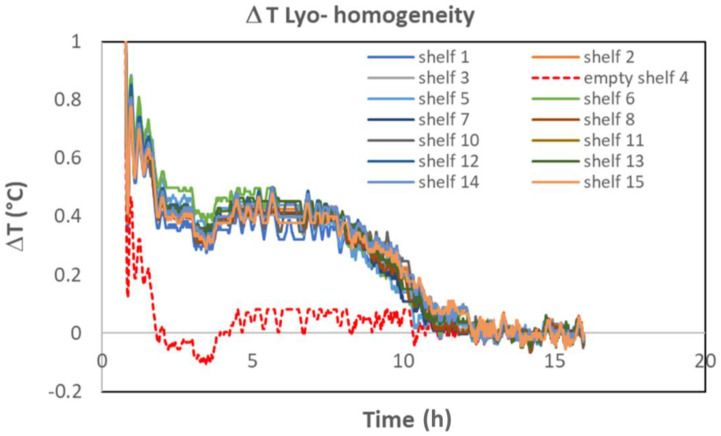
Δ*T* recording for each of the 15 shelves of a commercial freeze dryer. Shelf 4 was purposely kept empty (technical trial). Note, these data are coming from the same set of data as Figure 6B. The small negative peak around 4 h corresponds to the slow decrease of the shelf temperature from −15 °C to −20 °C. In this specific case, the baseline was not subtracted (cf. Figure 1A).

Many commercial freeze dryers are equipped with a temperature sensor at the outlet of each shelf. The Δ*T* method can therefore be applied individually to each shelf to check if the total sublimation flow per shelf (or per vial if at least one shelf is partially loaded) is identical for each shelf. Figure 7 shows an example where one shelf (shelf n° 4) from 15 was purposely kept empty. The signal is nearly superimposable for all full shelves and very different for shelf n° 4. This result shows that this method is capable of investigating shelf-to-shelf variability. Finally, in the case of a small batch, for which only a few shelves are loaded, the method applied to each shelf is much more sensitive than the method applied to the averaged outlet temperature, as the sublimation Δ*T* signal would be diluted by the signal of the unloaded shelves.

### 4.5. Process Transfer

When a freeze-drying cycle is transferred from the laboratory to industry, or from one industrial site to another, it is important to ensure that the thermal history of the product is identical. The classic method for ensuring this is to measure the product temperature in a limited number of vials, e.g., center and edge vials at both scales. However, it is generally impossible to measure product temperature directly under GMP conditions. Many laboratory freeze dryers, on the other hand, have only one shelf temperature measurement—e.g., the inlet only—and a capacitive pressure measurement in the chamber. In such cases, it is impossible to apply the Δ*T*/Δ*P* methods in the laboratory.

**Figure 8:**
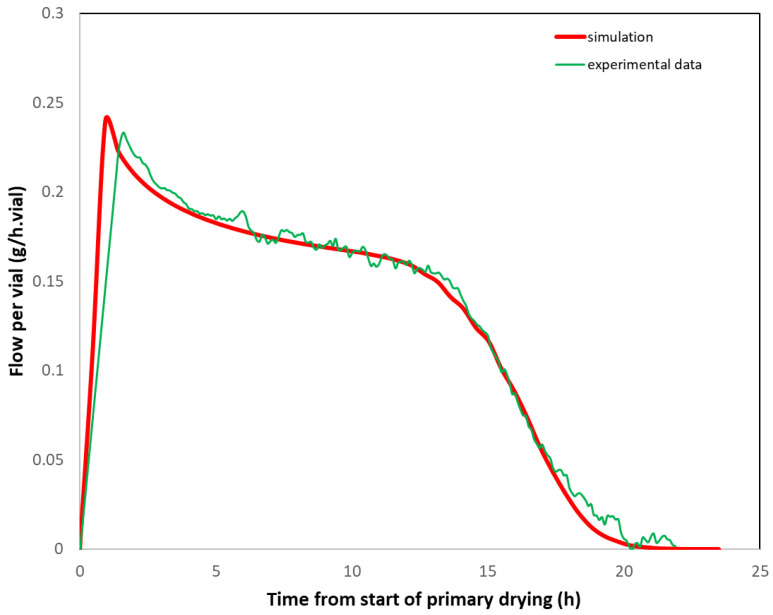
Comparison of the simulated sublimation flux per vial (g/h.vial) based on lab scale data (*Kv’s*, Rp) and the measured sublimation mass flow at pilot scale (Δ*T* method). Adapted from reference [17].

One method consists of measuring (i) the *Kv* distribution of the vials from ice test at the laboratory scale (e.g., using the method explained in reference [18]) and (ii) the cake resistance (Rp) from lab-scale product trials. Based on these data, it is possible to simulate [17] the total sublimation flux per vial for the reference cycle. The simulated sublimation flux (per vial) is then compared to that measured at large scale according to the Δ*T* or Δ*P* method. The two fluxes would be superimposed if the thermal history were identical on both scales. Since large-scale *Kv’s* are often smaller than laboratory *Kv’s* [21,22], it may be necessary to increase the large-scale shelf temperature by a few degrees to compensate. An example from reference [17] is provided in Figure 8. In this example, the lab scale trials were performed with vials in direct contact with the shelf (Martin Christ Epsilon-6D), whereas for the GMP batch (Usifroid 3 m^2^ pilot scale), vials were loaded on trays, resulting in significantly lower *Kv’s* (~40% decrease). Therefore, based on simulation calculations, the shelf temperature was raised from −5 °C at the lab scale to +5 °C at the pilot scale to compensate for the additional heat transfer resistance due to the tray. Figure 8 shows the good agreement between the simulated sublimation mass flow per vial corresponding to the lab scale. If the lab scale freeze dryer is equipped with a TDLAS probe or with chamber and condenser pressure probes, it is possible to directly compare the measured mass flow at both scales.

## 5. Ice Crystallization

**Figure 9:**
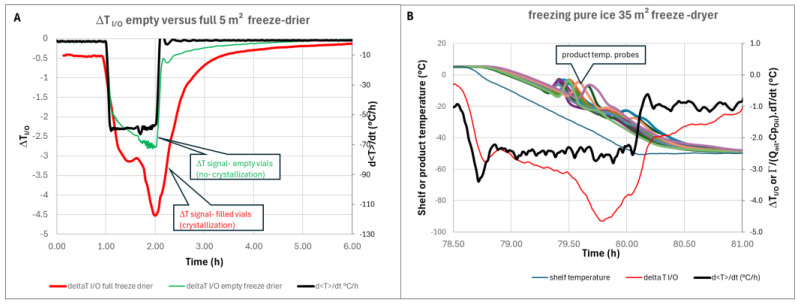
(**A**) Zoom on freezing (Δ*T* curve of empty freeze dryer is superimposed for comparison)—5 m^2^ pilot scale; (**B**) Δ*T* (Red) and d<T>/dt (black) superimposed with shelf temperature profile (dark blue) and temperature probes recording (all other curves)-35 m^2^ industrial scale.

Ice sublimation is a highly endothermic phenomenon (ΔH_s_ = 2836 kJ/kg), whereas ice crystallization is an exothermic phenomenon of much lower intensity (ΔH_c_ =−333 kJ/kg). On the other hand, while sublimation often lasts more than 24 h, crystallization usually takes place over a period of around one hour. Consequently, ice crystallization is accompanied by a sharp negative peak in Δ*T* (inlet–outlet), as shown in Figure 9A, because the fluid leaving the shelves is heated by the release of the crystallization enthalpy [14]. The comparison of the Δ*T* signal with product temperature probe recordings shows that the nucleation occurs much earlier in vials fitted with probes than in the general population, confirming that temperature probes accelerate nucleation and bias the observation [23] (Figure 9B). The shape of the Δ*T* signal is very similar to the shape of the heat flow measured by reference [3]. In this reference, the first small peak was interpreted as ice nucleation, whereas the second peak was interpreted as ice growth. An alternative interpretation may be that nucleation first occurs randomly in a select number of vials. The temperature within these vials rises close to zero very quickly, due to the enthalpy of ice crystallization. The temperature of the neighboring vials where nucleation did not take place increases due to vial-to-vial heat transmission. Therefore, nucleation is delayed in the neighboring vials, which explains the second peak [24]. The Δ*T* method provides an interesting alternative to product probes for the detection of nucleation.

## 6. Conclusions

Δ*P* and Δ*T* are non-invasive methods that offer a wide range of opportunities. Initially developed to measure ice sublimation flux using orthogonal and redundant methods, their use has been extended. For example, the Δ*T* method can be used to detect ice nucleation and crystallization. By combining sublimation flux measurement with modeling, it is possible to estimate the average product temperature and even the product temperature distribution. Finally, these methods allow us to assess the *Kv* distribution without having to carry out ice tests, which are labor-intensive and involve lengthy downtime, and are very costly for commercial units.

In practice, it is possible to use the average sublimation flux per vial as a criterion for transferring the freeze-drying cycle from the laboratory to commercial scale, or from one production plant to another during the product’s commercial life. The methods also make it possible to establish comparability between different freeze dryers, or even the homogeneity of sublimation from one shelf to another.

What these methods have in common is that they are extremely economical: no physical investment is required if the freeze dryers are equipped with inlet/outlet shelf temperature sensors (ideally one sensor per outlet of each shelf) and/or capacitive pressure sensors in the chamber and condenser. This is the case with most recent freeze dryers.

These methods, however, have their own limitations and challenges: they rely on the accuracy and sensitivity of existing temperature or pressure sensors (cross-calibration is solved by the baseline). The accurate estimation of the term $\frac{\Gamma }{Q_{oil}C_{p,oil}}\frac{d<T>}{dt}$ may be challenging and is freeze-dryer-specific.

Overall, despite their limitations, these methods are helping to speed up the development of freeze-drying cycles, improving understanding and hence robustness, while reducing costs. Future improvements may include improved mathematical data treatment through dedicated software and the involvement of equipment suppliers to improve the sensors’ sensitivity or to add silicone oil flow sensors. Ultimately, one may hope to use, for instance, the Δ*P* signal to determine in real time the mass flow and use it to control the freeze dryer.

## Data Availability

Not applicable.

## Nomenclature

The following abbreviations and symbols are used in this manuscript:

**Abbreviations**	
GMP	Good manufacturing practices
PAT	Process Analysis Technology
QbD	Quality by Design
TDLAS	Tunable Diode Laser Absorption System
**Symbols**	
Av	Area of vial outer section (m^2^)
C_p,oil_	Oil specific heat capacity (J/kg.°C)
dM/dt	Total sublimation or crystallization rate (kg/h)
*Kv*	Shelf to vial heat transfer coefficient (W/m^2^.°C)
M, m	Mass of ice in a vial, total mass of ice (kg,g)
P_chamber_	Chamber capacitance pressure (µbar)
P_condenser_	Condenser capacitance pressure (µbar)
<P>	Chamber, condenser average capacitance pressure (µbar)
Q_oil_	Silicone oil flow in the shelves (kg/h)
Rp	Cake resistance (m/s)
T_b_	Product bottom temperatures (°C)
T_s_	Shelf temperature (°C)
ΔH_s_, ΔH_c_	Enthalpy of water phase transition, sublimation, or crystallization (J/kg)
Δ*T*_I/O_	Shelf liquid inlet/outlet difference (°C)
G	Overall combined heat capacity of the shelves and vials: glass + water or ice (J/°C)

## Appendix A

**Table A1:** Equipment used and a succinct description of the experimental conditions.

Freeze Dryer	Example
Optima CS-5 5 m^2^, 6 shelves	Figure 1: the freeze dryer was fully loaded by 20R vials filled with 6.2 mL of 8% sucrose placebo. During primary drying, the pressure was set to 133 µbar and the shelf temperature to −5 °C.
	Figure 2: This example compares the correlation <P>Δ*P* vs. mass flow determined by Δ*T* method for several experiments. (1) The freeze dryer was fully loaded by 2R, 6R, or 10R vials containing pure water (2R, 6R) or an 8% sucrose solution (10R). During primary drying, the pressure was set to 133 µbar and the shelf temperature to −5 °C. (2) Test with 10 R vials in same conditions as (1), but with reduced number of vials (2/6 shelves). (3) Condenser test: 6 trays of water covering the complete shelves (89 kg of water) were loaded in the freeze dryer, and a complete freeze-drying cycle was performed. During primary drying, the pressure was set to 100 µbar. The shelf temperature was raised from −45 °C to 20 °C in 1.5 h and maintained at 20 °C for 65 h, until the trial was stopped after full sublimation of the ice.
	Figure 9A: freezing experiment. The freeze dryer was fully loaded with 20 R vials filled with 10 mL of pure water. The vials initially equilibrated at 0 °C were brought to −45 °C in 1 h. The temperature was then kept at −45 °C for more than five hours.
USIFROID SMH 90 3 m^2^ 5 shelves	Figure 8: full load of 10 R vials, undisclosed formulation. During primary drying, the pressure was set to 133 µbar and the shelf temperature to +5 °C. Vials were loaded on trays.
IMA Lyomax 29 29 m^2^, 13 Shelves	Figure 3: refers to the mass flow measurement according to the Δ*P* and Δ*T* methods during the primary drying of the commercial product “A”. Further information is not disclosed.
	Figure 6A: refers to a technical run of the commercial product “B”. During this non-GMP run, Ellab temperature probes were used to monitor the product temperature. The shelf temperature profile during primary drying is indicated in the figure. Further information is not disclosed.
IMA Lyofast 35 35 m^2^, 15 shelves	Figure 5A,B, Figure 6B and Figure 7 are related to the same technical run of an undisclosed placebo formulation: 14 of the 15 shelves of the freeze dryer were filled with 2R vials containing 1 mL of an undisclosed formulation. During the primary drying, the pressure was set to 60 µbar and the shelf temperature profile is disclosed in the figure. During this non-GMP run, Ellab temperature probes were used to monitor the product temperature.
	Figure 9B: freezing part of lyophilization cycle. 142000 2R vials filled with 1 mL of water were loaded in the freeze dryer (qualification run). The vials were first equilibrated at 5 °C, then the shelf temperature was brought to −45 °C in about 1.2 h. The temperature was kept at −45 °C for several hours.
